# An unexpected finding in a patient with a DDDR-PM

**DOI:** 10.1007/s12471-014-0552-x

**Published:** 2014-04-04

**Authors:** A. Bohm, R. G. Kiss, G. Z. Duray

**Affiliations:** Department of Cardiology, Military Hospital, Róbert K körút 44, Budapest, 1134 Hungary

**Fig. 1:**
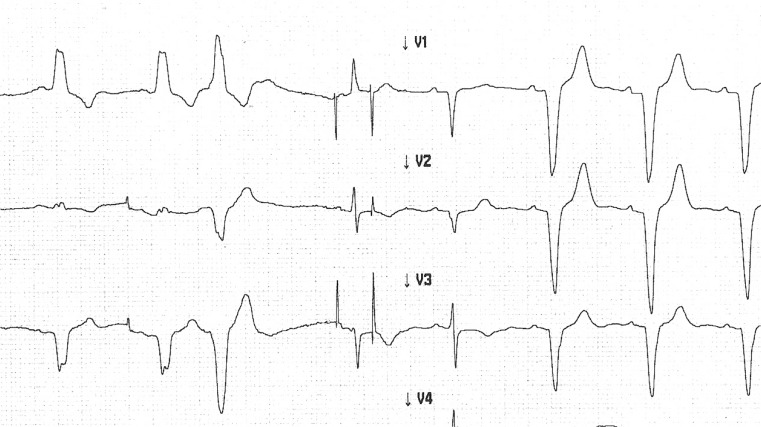
Ventricular sensing failure, when the QRS is narrow

We noticed an unprecedented sensing error: after a correctly sensed ventricular extrasystole followed by a correctly timed atrial stimulus, a ventricular impulse occurs that is not sensed, then the delivery of a ventricular stimulus can be observed at the set AV delay (225 ms) (Fig. 1). The subsequent stimuli appear to be sensed correctly, judging from the lack of any atrial or ventricular spikes.

**Fig. 2:**
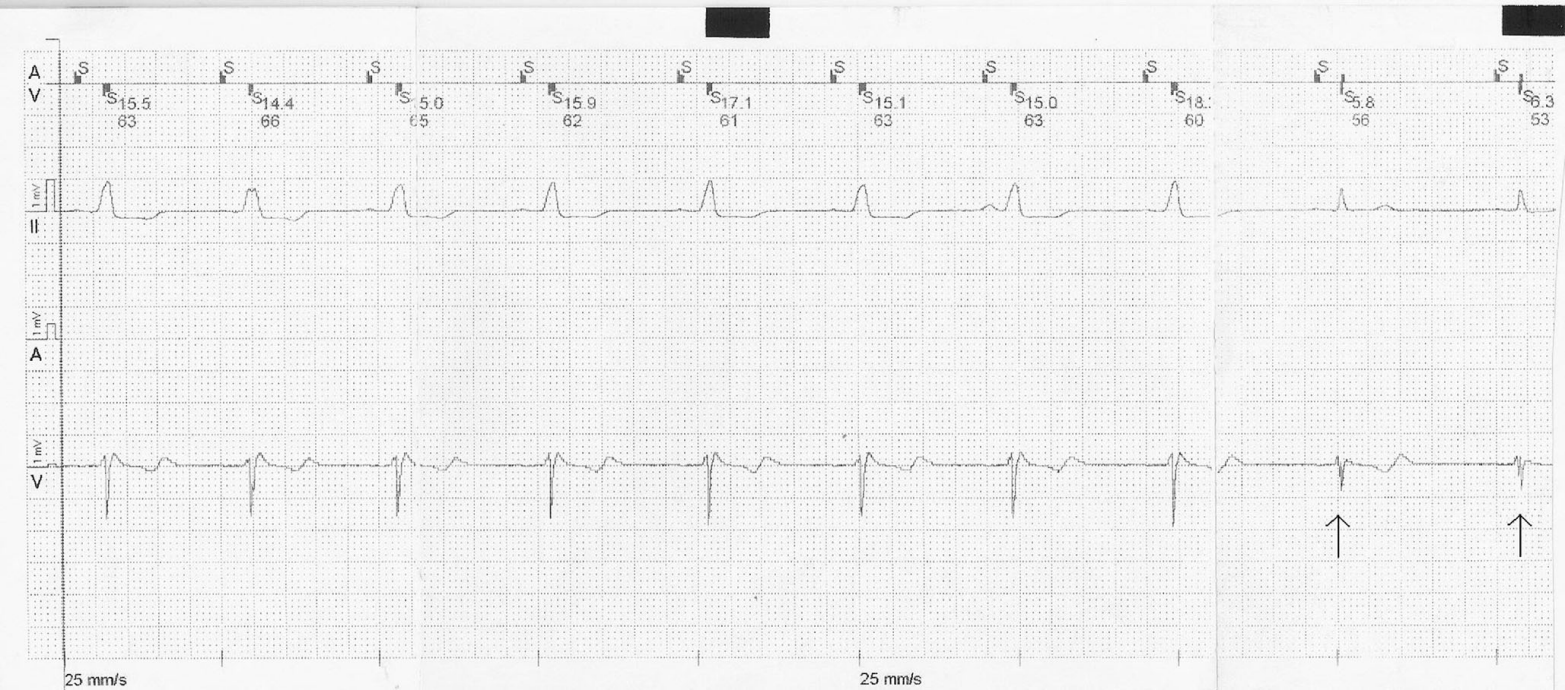
Bundle branch dependent R wave amplitude

IEGM scans eventually revealed the cause of the unexpected sensing error: while the R-wave amplitude exceeded 15 mV with a bundle branch block, we sensed a significantly lower R-wave amplitude with a narrow QRS (5.8–6.3 mV in the last two QRS) (Fig. 2). At a sensitivity of 5 mV, the pacemaker only intermittently sensed a narrow QRS. We have now lowered the ventricular sensitivity to an appropriate level.

